# A Modular Platform for Porphyrin Molecular Design and UV–Vis–NIR Spectral Prediction

**DOI:** 10.3390/molecules31173052

**Published:** 2026-08-31

**Authors:** Qirui Sun, Hanchi Zhao, Guofan Zhang, Jiaao Han, Rui Liu

**Affiliations:** 1 Division of Natural and Applied Sciences, Duke Kunshan University, Kunshan 215316, China; 2 Digital Innovation Research Center, Duke Kunshan University, Kunshan 215316, China

**Keywords:** porphyrin, absorption spectra, photochemistry, fluorophores, software, website, web design, Gouterman model

## Abstract

Porphyrin and related tetrapyrrole chromophores underpin broad applications in photochemistry, photomedicine, catalysis, and functional materials, yet practical workflows that connect molecular design, geometry optimization, orbital-energy prediction, and absorption spectrum prediction remain fragmented across separate computational tools. Here, we report BeyondPhoton, a modular web- and software-based platform that enables automated porphyrin molecular design and electronic absorption spectral prediction within a single continuous workflow. Four interconnected but independently operable Flask applications communicate through lightweight JSON data exchange, allowing users to proceed from de novo molecular construction or direct SMILES input to geometry optimization, structural visualization, frontier molecular orbital energy prediction, and UV–Vis–NIR spectral simulation without manual file conversion. Predicted HOMO−1, HOMO, LUMO, and LUMO+1 energies are translated into B_y_, B_x_, Q_y_, and Q_x_ transitions using the Gouterman four-orbital model with empirical correction and Gaussian broadening. Interactive visualization, automated peak annotation, and batch-exportable data formats support rapid screening and downstream analysis. This integrated platform organizes porphyrin spectral prediction into transparent modules, reducing workflow fragmentation and providing a scalable computational infrastructure for tetrapyrrole molecular design in photoscience applications.

## 1. Introduction

With the rapid advancement of artificial intelligence and data-driven methods, a growing number of machine learning tools for molecular property prediction have emerged in computational chemistry and molecular photonics. Recent studies have demonstrated machine learning prediction of porphyrin molecular orbital energy gaps from molecular graph representations [1] and quantitative structure–property relationships for porphyrin photochemistry, including singlet-oxygen generation [2]. Broader reviews of machine learning methods in photochemistry/photophysics and of QSPR/ML applications in molecular photonics further establish the context for such approaches [3,4]. These tools aid conventional experimental approaches with computational predictions, thereby reducing research costs and shortening development cycles. Although existing computational and predictive tools are generally user-friendly, most of them are designed to perform isolated functions and lack integration with complementary platforms [5,6,7,8]. In addition, heterogeneity in input and output data formats across different tools complicates data exchange and hinders seamless interoperability between computational stages. This fragmentation increases operational complexity, complicates research workflows, and ultimately diminishes the overall efficiency of computational prediction efforts.

**Figure 1:**
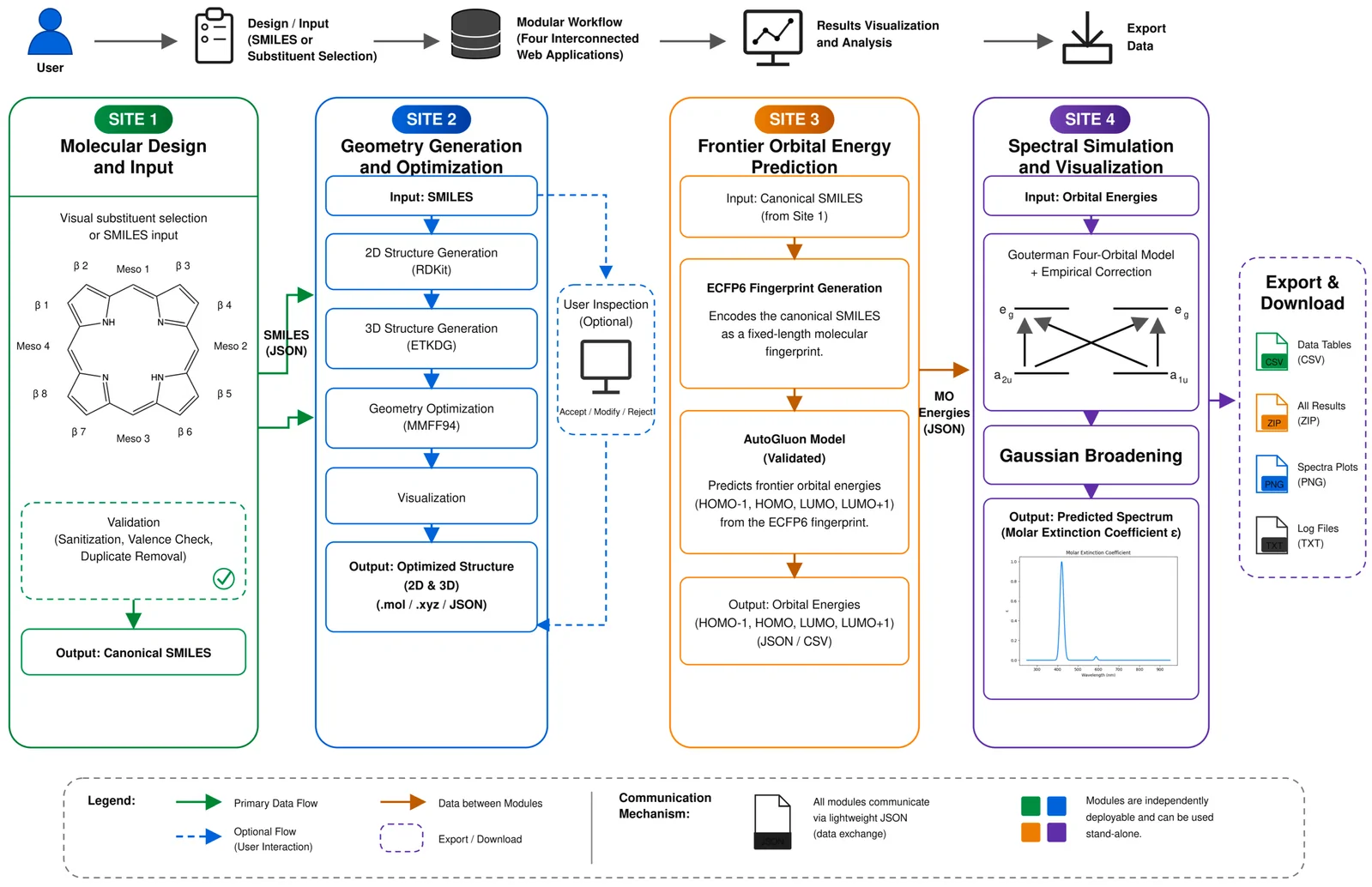
System architecture and complete workflow of the four-site BeyondPhoton platform. Site 1 generates canonical SMILES from visual substituent selection or SMILES input after validation. Site 2 performs optional RDKit/ETKDG embedding and MMFF94 geometry optimization with 2D/3D visualization (optional user inspection). Site 3 predicts HOMO−1, HOMO, LUMO, and LUMO+1 energies (GFN2-xTB and/or the validated machine-learning model) and transfers them to Site 4. Site 4 applies the Gouterman four-orbital model with empirical correction and Gaussian broadening to produce the predicted UV–Vis–NIR spectrum. Solid arrows indicate primary data flow; dashed arrows indicate optional user-interaction paths. Modules communicate via lightweight JSON and can operate independently.

To address the limitations arising from such fragmentation, we developed BeyondPhoton, a modular multi-site web and desktop platform built with the Flask framework [9]. The system consists of four interconnected sites that perform molecular structure construction, geometry optimization, molecular orbital energy prediction, and spectral simulation, respectively. Together, these sites constitute a fully automated workflow that enables uninterrupted data transfer, while each site can also operate independently as a stand-alone tool. Specifically, Site 1 provides a porphyrin molecular design interface that allows users to introduce substituents at the meso and β positions (Figure 1), thereby generating the corresponding SMILES representations of the designed molecules [10]. These SMILES strings can be transmitted to Site 2 and Site 3. Site 2 generates both two-dimensional and three-dimensional visualizations of the molecular structures, allowing users to verify the resulting porphyrin architectures. Site 3 converts sanitized SMILES into ECFP6 fingerprints and applies a previously trained AutoGluon ensemble to predict frontier molecular orbital energies (HOMO−1, HOMO, LUMO, and LUMO+1) [11,12,13]. Finally, the outputs from Site 3 are forwarded to Site 4, where the data are transformed into visualized absorption spectra based on the Gouterman four-orbital model [14,15,16,17,18] and further refined through Gaussian broadening and empirical correction to produce intensity-normalized predicted spectra.

In contrast to conventional computational programs that often suffer from fragmented prediction workflows, the present system implements four independent but data-connected sites to coordinate and streamline the overall prediction workflow [19]. Each site is responsible for data conversion, computation, and prediction tasks specific to its functional module, while inter-site data exchange enables full automation of the workflow. The proposed architecture allows the system to operate either as an integrated workflow or as a collection of stand-alone tools for specific tasks. Under this modular framework, each step of the prediction process remains transparent and traceable, thereby ensuring reproducibility and flexibility for subsequent modification.

In this work, we present a batch-capable, visualized, end-to-end workflow for porphyrin molecular design and property prediction. BeyondPhoton provides (i) batch visualized molecular design with automatic validation and canonical SMILES generation; (ii) batch generation of initially optimized 2D/3D structures with per-molecule optimization status; (iii) batch prediction of HOMO−1/HOMO/LUMO/LUMO+1 energies and conversion into B/Q transitions and continuous UV–Vis–NIR spectra; and (iv) continuous, traceable data transfer between modules through standardized lightweight data structures. These functions transform previously disconnected molecular-generation, structural-processing, machine learning, and spectral-simulation scripts into an integrated research workflow for batch screening of porphyrin candidates.

To support research in computational chemistry and to promote a research environment centered on reproducible and network-based computation, the modular system described herein has been designed and deployed as outlined in Figure 1.

## 2. Results

### 2.1. System Overview and Entry Methods

**Figure 2:**
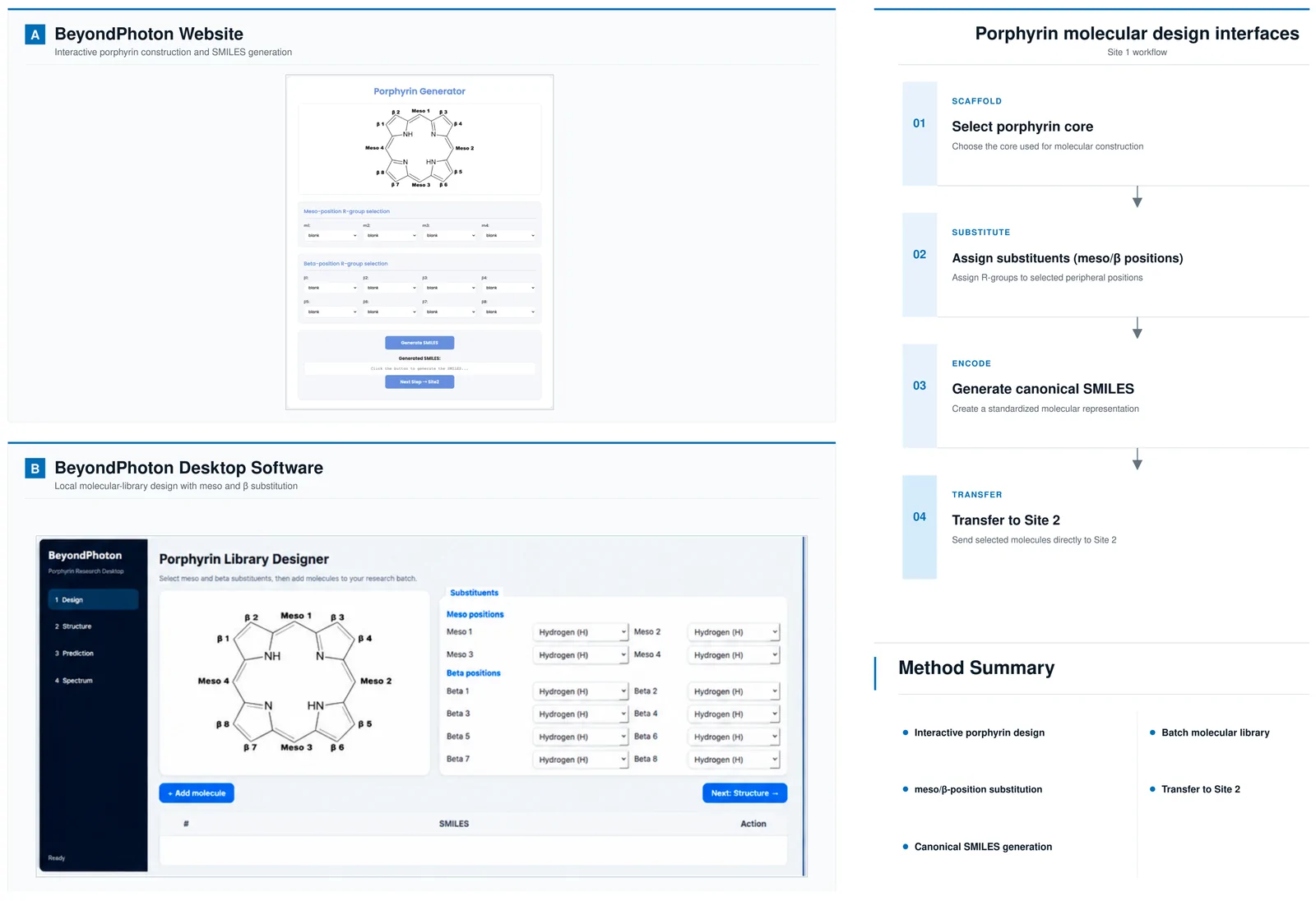
Porphyrin molecular design interfaces in BeyondPhoton: (**A**) Website Porphyrin Generator with labeled meso and β positions and R-group selectors. (**B**) Desktop Porphyrin Library Designer. Site 1 workflow proceeds from scaffold selection to substituent assignment, canonical SMILES generation, and transfer to Site 2.

The system adopts a visualized porphyrin substituent selection interface as its primary entry point. In this interactive Site 1 mode, molecular design is user-directed rather than random: the user explicitly selects substitution positions on the porphyrin macrocycle (meso and/or β) and assigns the desired substituent to each selected position. Site 1 then assembles the specified structure, verifies chemical validity, and generates the corresponding canonical SMILES (Figure 2). Alternatively, if a target SMILES is already available, Site 1 can be bypassed, and the string entered directly into Site 2 for structural visualization. Once the structure displayed in Site 2 is verified, the same SMILES can be submitted to Site 3 for orbital-energy prediction and then to Site 4 for spectral calculation. This flexible workflow enables a more efficient and streamlined prediction process (Figure 2).

### 2.2. Text-Based SMILES Input Workflow

Users can input one or more SMILES strings in Site 2 (Table 1). The system supports two input methods: (1) direct text input in the text area or (2) drag-and-drop upload of Excel files (.xlsx or .xls files). When an Excel file is dragged into the input area, it will be automatically uploaded and processed. The Excel file should contain columns for order and SMILES for each molecule. The system will first perform legality checks on each input, automatically complete standardization, and then send it to the geometry optimization module. If there are problems with the SMILES strings, the system will indicate the specific content of the problem. If the user performs batch processing, the system will record the processing status of each molecule. The uploaded file information is displayed visually on the page, showing the file name and size with an option to remove the file if needed.

### 2.3. Visual Substituent Selection Workflow

The interactive substituent selection interface provides a fully labeled unsubstituted porphyrin parent core, and users can select substituents at meso and/or β positions. The system instantly generates the corresponding porphyrin SMILES from the internal library and performs legality verification of the generated structure. This user-controlled constructor is complementary to the meso-only automated batch generator in Section 4.1. Table 2 lists the substituent categories available in Site 1.

### 2.4. 2D and 3D Molecular Display

**Figure 3:**
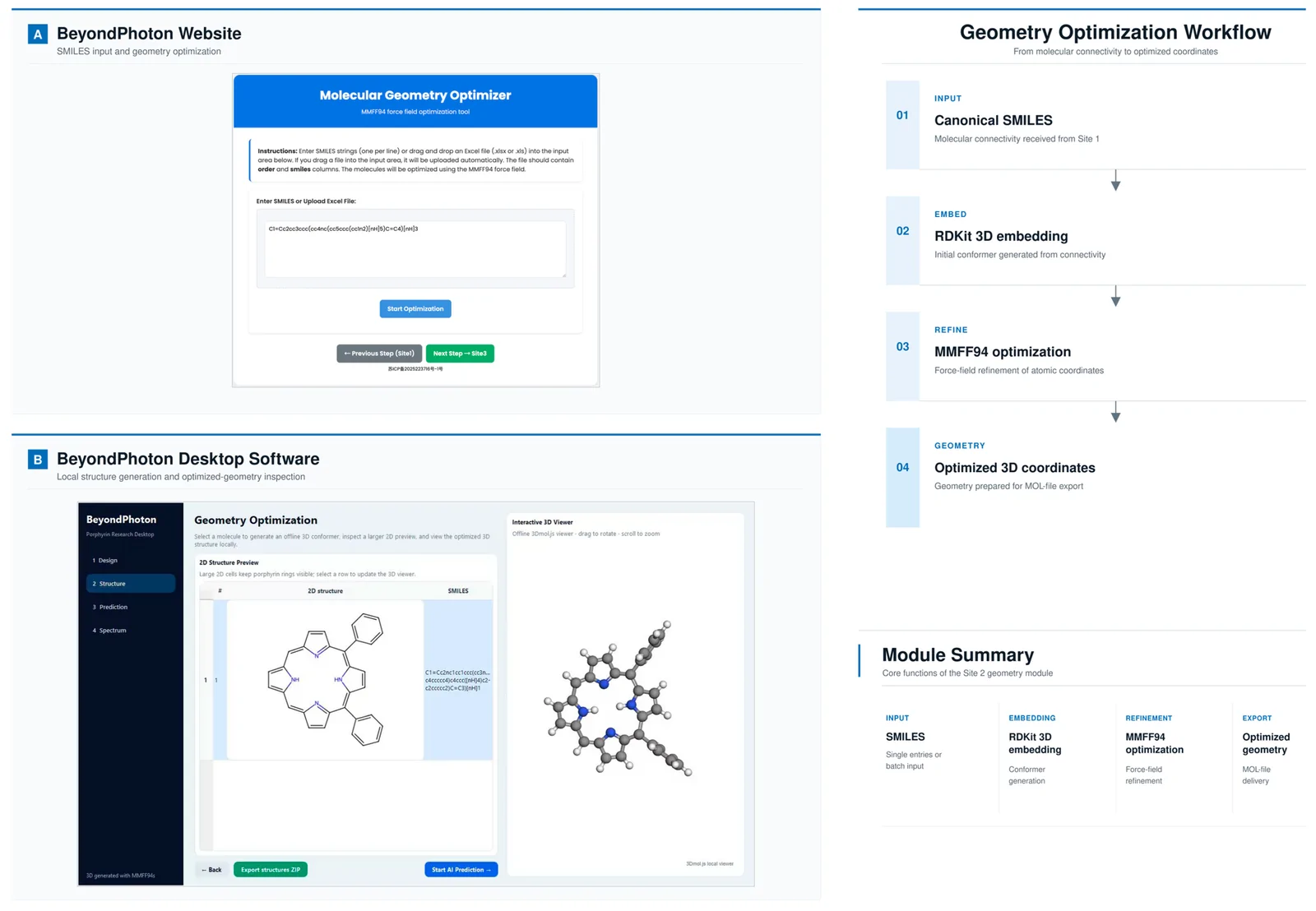
Molecular geometry optimization interfaces in BeyondPhoton: (**A**) Website Molecular Geometry Optimizer accepting single or batch SMILES (or spreadsheet upload) for MMFF94 optimization. (**B**) Desktop geometry-optimization module with 2D preview and interactive 3D viewer. Site 2 workflow converts canonical SMILES to RDKit 3D embedding, MMFF94 refinement, and exportable optimized coordinates. (The Chinese text in the website footer (also visible in Figures 4–7) is the statutory China ICP (MIIT) website filing identifier required for the deployed BeyondPhoton web service; it is not a scientific label). Note: The remaining website results, including the 2D structure preview, interactive 3D viewer, and export buttons, are shown in Figure 5.

The structure optimized by Site 2 is presented simultaneously as an RDKit-generated 2D PNG image (600 × 600 pixels; rdCoordGen) and a 3D model rendered with 3Dmol.js [20]. Default geometry generation uses RDKit distance-geometry embedding followed by MMFF94 minimization for supported organic atom types. Multiple conformers may be generated with distinct random seeds; structures are pruned by an RMS threshold, and the lowest-MMFF94-energy conformer is retained for display/export. Force-field convergence is judged by the MMFF94 energy/gradient criteria and a maximum iteration limit; failure triggers fallback embedding with an increased random seed or alternate conformer-search parameters. Distance-geometry embedding plus MMFF94 identifies a local minimum for each starting conformer and does not guarantee the global minimum. Each optimized molecule is displayed as “Set X” with per-molecule MOL/PNG downloads and batch export options (Figure 3).

### 2.5. Molecular Orbital Energy Prediction

**Figure 4:**
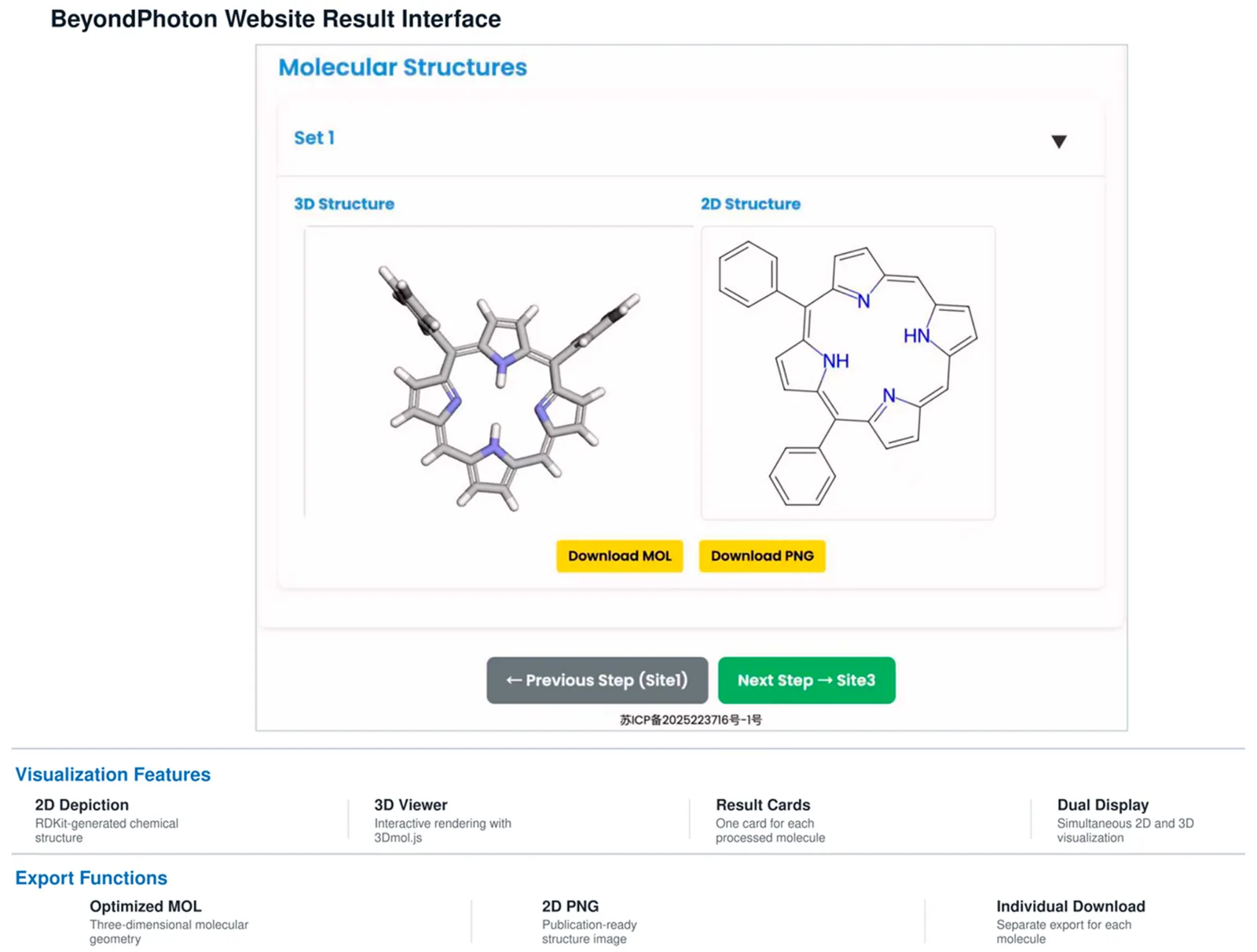
BeyondPhoton Site 2 result interface showing simultaneous 3D (3Dmol.js) and 2D (RDKit) structural visualization for an optimized molecule, with per-molecule MOL/PNG download options and navigation to Site 3.

Valid SMILES inputs are used by Site 3 to predict the energies of the four frontier molecular orbitals. An invalid SMILES in this workflow is a string that cannot be parsed into a chemically consistent RDKit molecular graph or that fails validation because of impossible valence, impossible ring formation, unsupported bonding, or failed fragment attachment. After parsing and sanitization, Site 3 generates ECFP6 fingerprints (radius 3; 2048 bits) and applies an AutoGluon ensemble previously trained to predict HOMO−1, HOMO, LUMO, and LUMO+1 energies [11,12,13]. AutoGluon is an automated machine-learning framework for structured data that trains, tunes, ensembles, bags, and stacks multiple candidate learners under a specified evaluation objective; in the prior model, it was used to select and combine regressors for the four orbital-energy targets [13]. Site 3 does not perform a semiempirical quantum-chemical calculation. Predicted results are tabulated with orbital labels, energies, and status flags. From the prior Scientific Reports study on 1437 metal-free porphyrins, chlorins, and bacteriochlorins, the reported validation metrics are R2 = 0.985, MAE = 0.077 eV, and RMSE = 0.133 eV, with held-out test R2 = 0.936, MAE = 0.168 eV, and RMSE = 0.287 eV [12]. These metrics characterize the underlying orbital-energy model reused by the platform and are summarized here to avoid duplicating the full prior validation (Figure 4).

**Figure 5:**
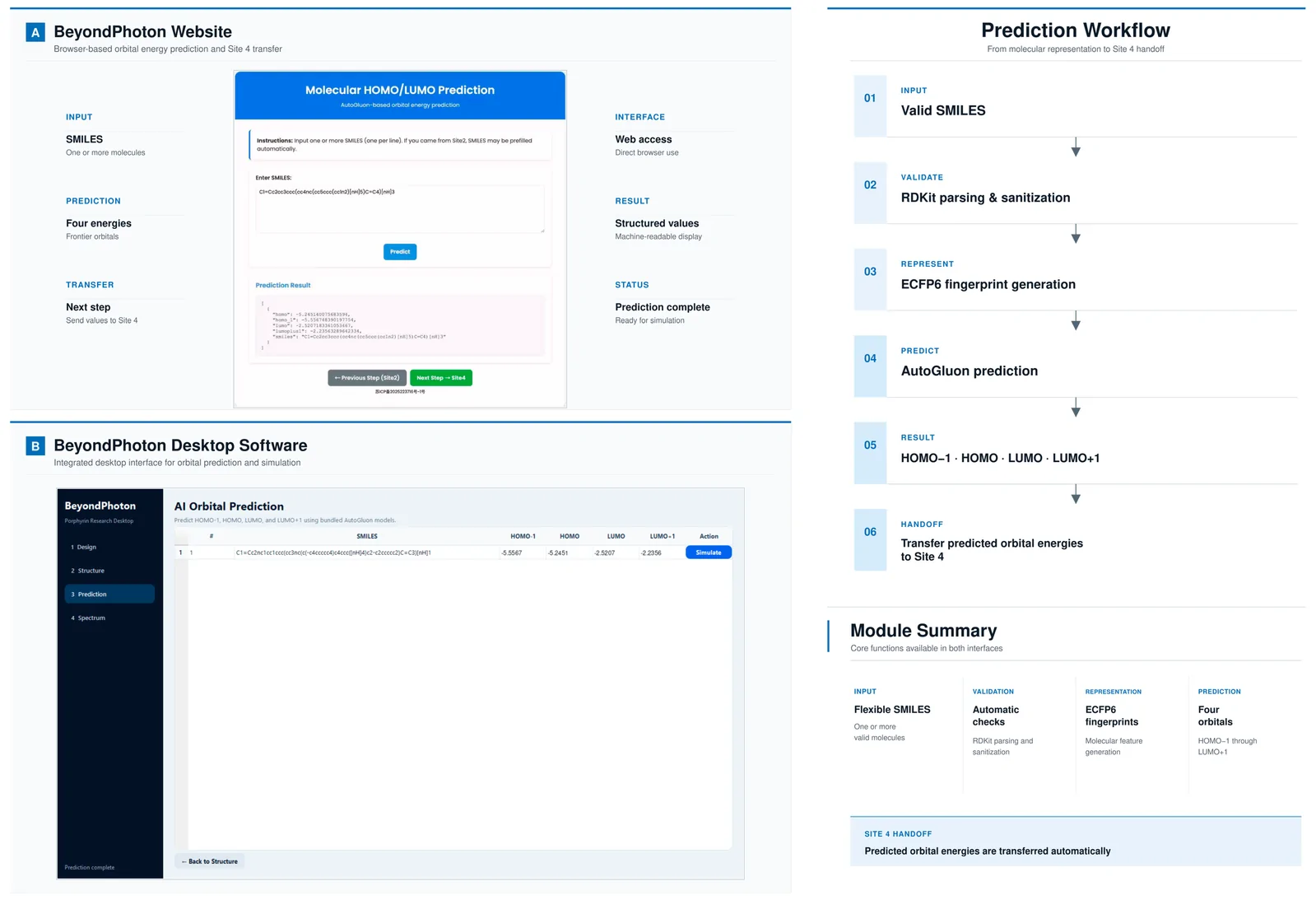
Frontier-orbital energy prediction in BeyondPhoton: (**A**) Website AutoGluon-based HOMO/LUMO prediction from SMILES with machine-readable JSON output. (**B**) Desktop AI Orbital Prediction module. The workflow validates SMILES, generates ECFP6 fingerprints, predicts HOMO−1, HOMO, LUMO, and LUMO+1 energies, and hands off values to Site 4. Predicted orbital energies are used under the working assumption that the frontier orbitals retain ligand-centered a1u/a2u and eg-derived nodal patterns of the Gouterman four-orbital model; strongly perturbing substituents may invalidate that assumption (see Discussion).

Table 3 summarizes these prior orbital-energy validation metrics for the Site 3 model; they are reused by BeyondPhoton and are not re-derived in the present platform study (Figure 5).

### 2.6. Spectrum Calculation, Normalization, and Peak Reporting

**Figure 6:**
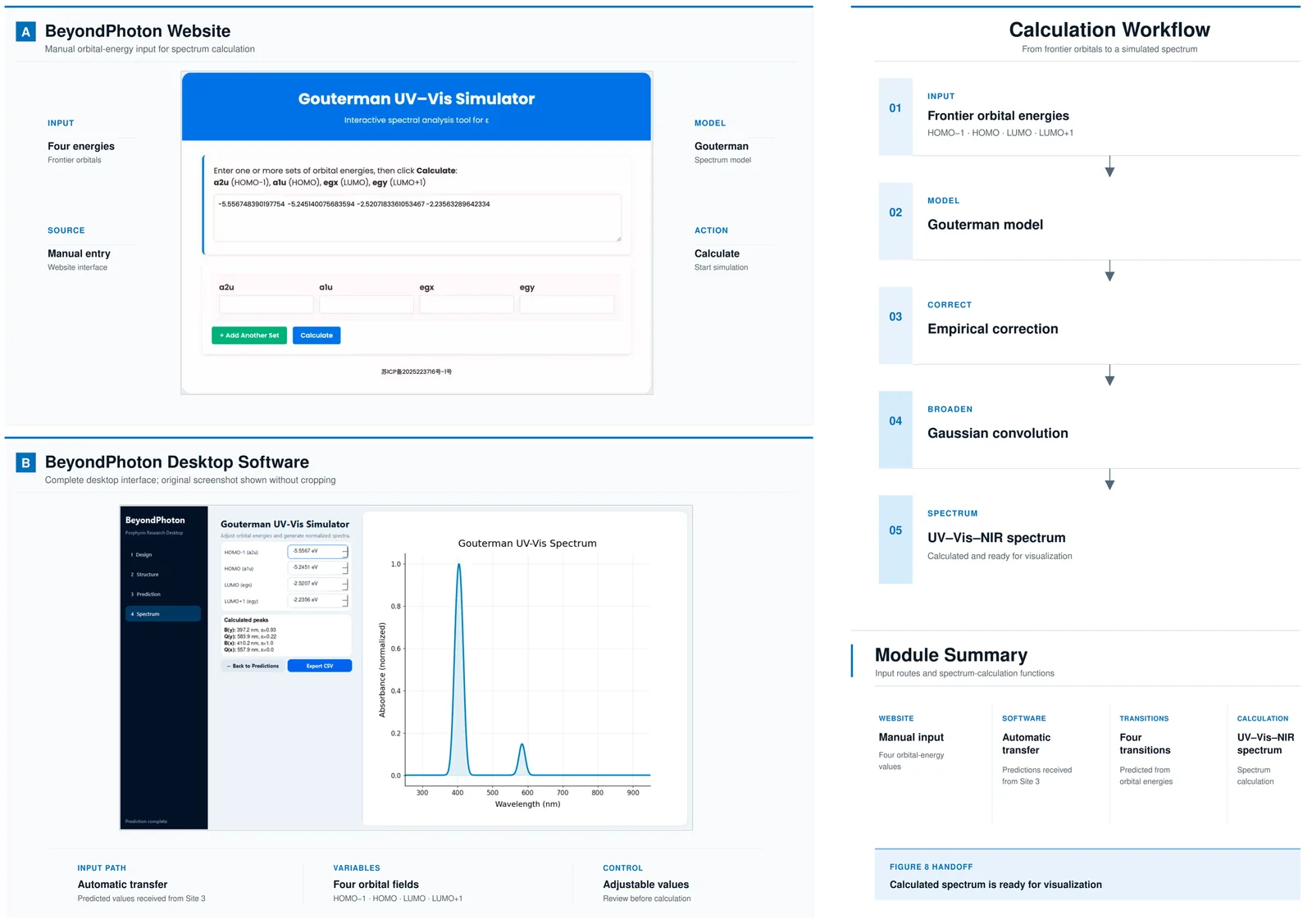
Site 4 orbital-energy input and spectral handoff: (**A**) Website Gouterman UV–Vis simulator with manual entry of four frontier orbital energies. (**B**) Desktop Spectrum module with automatic transfer of Site 3 predictions, calculated as By/Bx/Qy/Qx peaks, and normalized spectrum display. The calculation workflow applies the Gouterman model, empirical correction, and Gaussian broadening.

Site 4 generates four optical transition energies of the target porphyrin molecule that correspond to its By, Bx, Qy, and Qx transitions, respectively, based on the orbital energies from Site 3 and produces the complete absorption spectrum through empirical correction and Gaussian convolution (Figure 6). The structure of the peak value table output by the system is as follows (Table 4).

### 2.7. Performance Evaluation and Case Study

**Figure 7:**
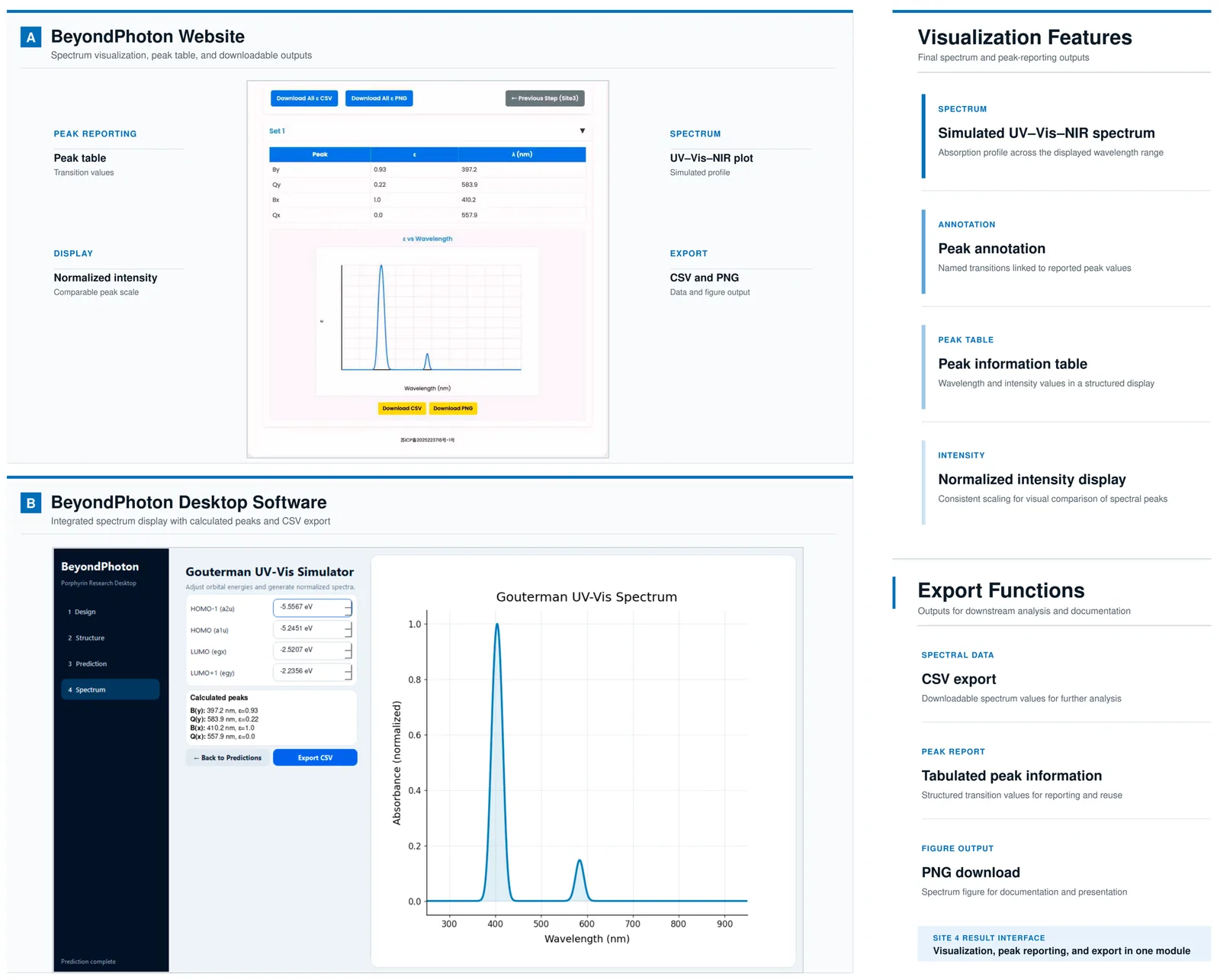
Site 4 spectral result interface: (**A**) Website peak table and normalized UV–Vis–NIR plot with CSV/PNG download. (**B**) Desktop integrated spectrum display with orbital-energy controls, annotated peaks, and CSV export. Intensities are normalized; wavelength remains in physical nanometers.

**Figure 8:**
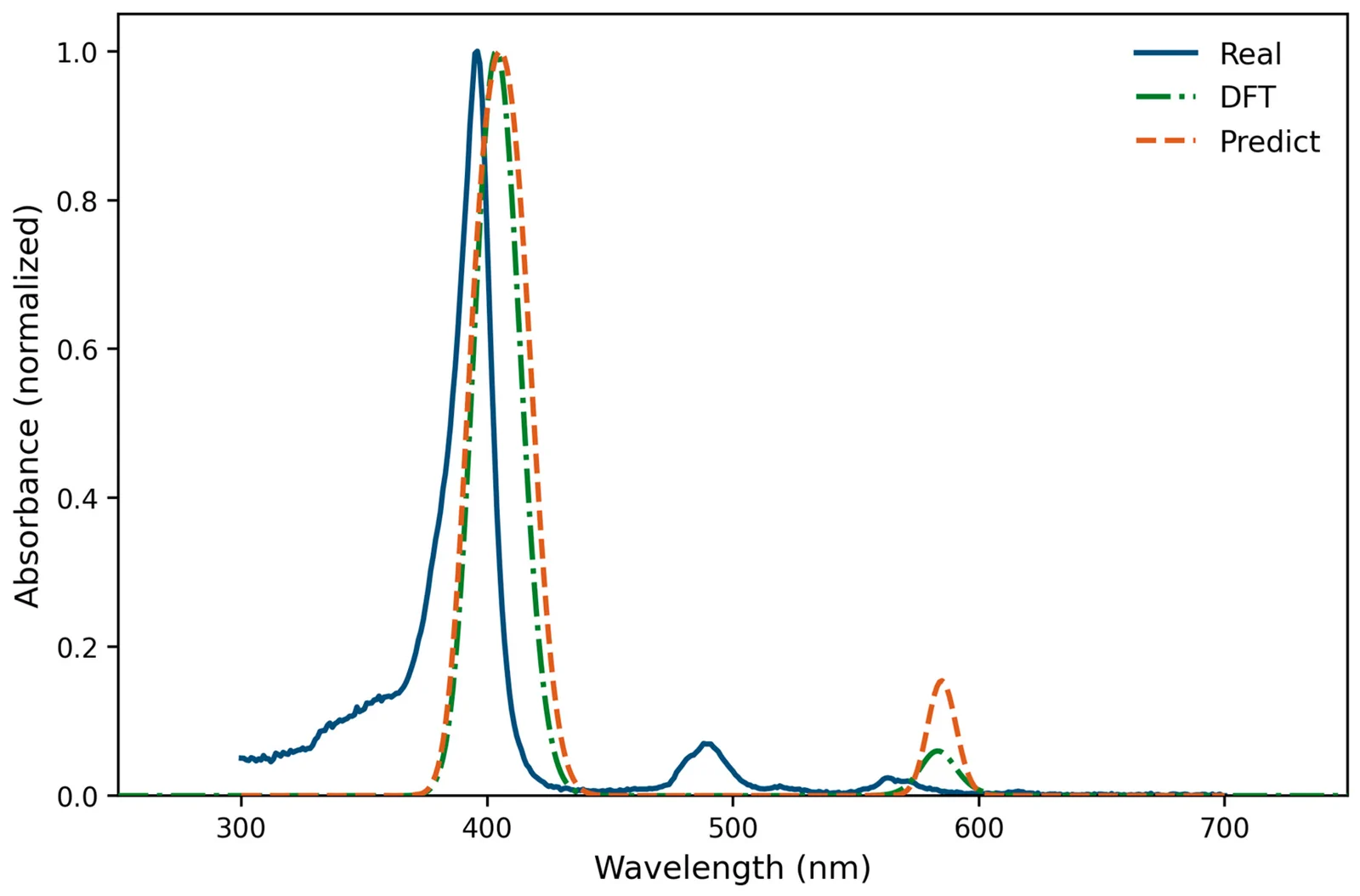
Comparison of experimental, platform-predicted, and DFT-simulated normalized absorption spectra of 5,10,15,20-tetraphenylporphyrin (TPP). Blue solid line: experimental (Real) spectrum; orange dashed line: BeyondPhoton prediction; green dash-dotted line: DFT-based simulation. The experimental Soret maximum is at 396 nm; BeyondPhoton and DFT B-band maxima appear at 405 and 404 nm, respectively. The experimental feature near ~480–488 nm is not reproduced by the four-transition electronic-region model, consistent with the absence of explicit vibronic coupling and solvent effects in the current simulation.

To evaluate the predictive performance of the integrated BeyondPhoton platform against experimental data and higher-level electronic-structure simulations, we selected 5,10,15,20-tetraphenylporphyrin (TPP) as a representative benchmark chromophore. Normalized experimental (Real), BeyondPhoton-predicted, and DFT-simulated absorption spectra were compared over the UV–Vis region (Figure 7 and Figure 8). The experimental spectrum shows an intense Soret (B) maximum at 396 nm (3.131 eV), together with weaker Q-region features (including a band near 488 nm and a local maximum at 563 nm). The BeyondPhoton continuous spectrum places the dominant B-band maximum at 405 nm (3.061 eV; Δλ = +9 nm relative to experiment) and the principal Q-band maximum at 585 nm (2.119 eV). The DFT-based simulation yields a B maximum at 404 nm (3.069 eV; Δλ = +8 nm) and a Q maximum at 583 nm (2.126 eV), closely tracking the platform region. Absolute peak positions and photon energies are summarized in Table 5. Neither the present four-transition electronic-region model nor the DFT comparison used here reproduces vibronic progressions in the Q-band region; accordingly, the experimental feature near ~480–488 nm in Figure 8 is not recovered and is attributed to vibronic/solvent contributions outside the current electronic four-peak model. Mean absolute differences between normalized absorbance traces over 300–700 nm are 0.086 (experiment vs. BeyondPhoton) and 0.076 (experiment vs. DFT), underscoring that region-level agreement can remain moderate even when the main B/Q positions are chemically reasonable. Overall, this case study shows that BeyondPhoton delivers interpretable UV–Vis–NIR transitions consistent with the main experimental B/Q pattern for a prototypical free-base porphyrin, while remaining subject to the documented limitations of the underlying orbital-energy and four-orbital spectral models.

Additional PRE versus TRUE spectral-region comparisons for representative electron-withdrawing, electron-donating, and conjugated meso-substituted libraries (EWG/EDG/CONJ) were generated with the same Site 4 workflow and show similar B-band shifts on the order of ~8–10 nm for EWG and CONJ subsets, reinforcing that the platform is intended for rapid region-level screening rather than vibronically resolved peak matching.

## 3. Discussion

### 3.1. Target User Groups

This system is targeted at the broad research communities related to porphyrin photophysics and chemistry, including photocatalysis, photovoltaic materials, photomedicine, sensor chemistry, environmental photochemistry, and quantum materials. Since this system specifically handles porphyrins, chlorins, bacteriochlorins, and other macrocyclic systems derived from porphyrin molecules, the primary target group consists of researchers engaged in related fields. Considering that research in these fields is widespread globally, the system adopts a cross-platform-compatible strategy during design, and the step-by-step interface reduces the difficulty for new users when first using the website. Research in these communities is commonly disseminated through journals spanning photochemistry, physical chemistry, inorganic/organic chemistry, and materials science; representative outlets, grouped by publisher, are summarized in Table 6.

### 3.2. Literature Foundation

The theoretical foundations of this system cover porphyrin spectroscopy and the Gouterman four-orbital model [14,15,16,17,18]. Model validity requires that the four frontier orbitals retain ligand-centered a1u/a2u and eg-derived nodal patterns of the porphyrin π system (or the related a, s, −a, and −s labels in Michl’s 4N + 2 perimeter model); with strongly perturbing meso-aryl or electron-withdrawing substituents, these orbitals are not automatically guaranteed to be Gouterman’s four orbitals, and the software does not automate that classification [21,22,23]. Michl’s soft- and hard-chromophore terminology, based on the magnitudes of ΔHOMO and ΔLUMO and the extent of departure from accidental near-degeneracy of the parent HOMO pair, provides a useful language for stating which structural perturbations remain within the present spectral model [21,22,23].

Note that the present orbital-energy model is validated only for metal-free porphyrin, chlorin, and bacteriochlorin structures represented in the training data. Metalloporphyrins and structures containing unsupported heavy elements fall outside the validated domain. MMFF94 geometry generation is used only when atom types and parameters are available and should not be interpreted as a validated treatment of any metalloporphyrins. New models that are capable of predicting metalloporphyrins are under development. Likewise, extending the approach to phthalocyanines and many related analogues is not currently feasible because those families are outside the training domain.

Any prediction of vibronic overtone in the optical spectra is not provided by the current model and platform (and the TDDFT comparisons used here). Magnetic circular dichroism (MCD) spectroscopy is also not implemented; the current training dataset does not contain MCD observables. Future work will explore MCD-derived sign sequences and moment analyses as an independent test of orbital-energy ordering (ΔHOMO/ΔLUMO) following the foundational perimeter-model framework of Michl and coworkers [21,22,23].

### 3.3. User Feedback and System Adaptability

Although this project has not yet undergone formal email promotion or statistical data collection, based on the system function settings and usage scenarios, the anticipated user community is international and interdisciplinary. To adapt to different usage environments, the system design already ensures the following:

- It is compatible with desktop, mobile, and tablet devices;
- All pages can run in mainstream browsers;
- It supports multiple workflows from single molecules to batch molecules;
- All computational results can be exported and used for further scientific analysis.

Therefore, even without formal promotion, this system still possesses the potential to provide support for the porphyrin and tetrapyrrole research communities and can continuously expand its functions with user feedback.

## 4. Design and Methods

### 4.1. Automated Batch Generation of Porphyrinoid SMILES

**Figure 9:**
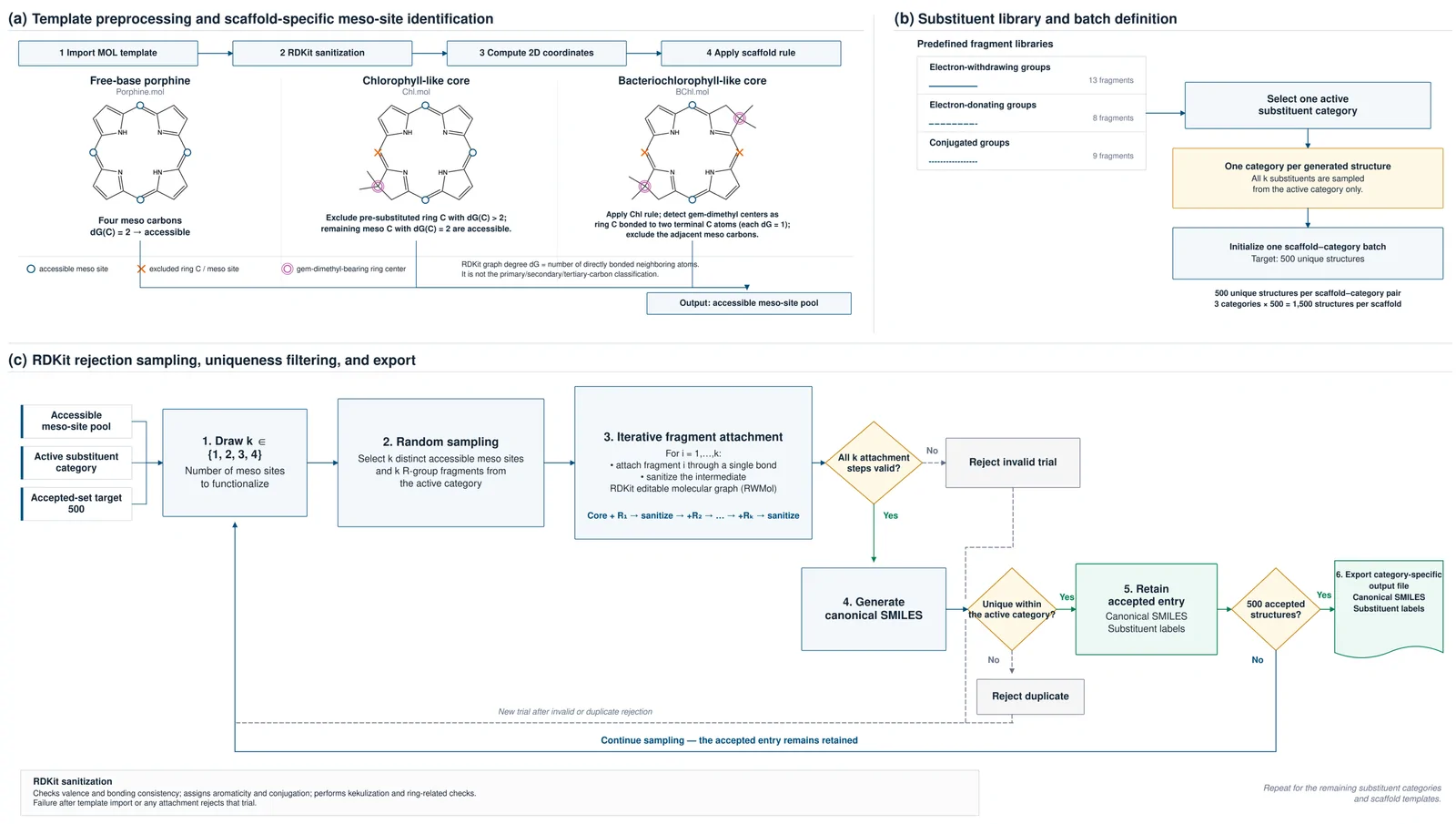
Workflow of automated batch generation of meso-substituted porphyrinoid SMILES: (**a**) Template preprocessing and scaffold-specific meso-site identification for free-base porphine, chlorophyll-like, and bacteriochlorophyll-like cores. (**b**) Substituent libraries grouped into electron-withdrawing, electron-donating, and conjugated categories. (**c**) RDKit rejection-sampling loop with sanitization, uniqueness filtering, and export of canonical SMILES plus substituent labels. Scope: automated stochastic meso-only batch generator; interactive meso/β construction is shown in Figure 3.

To construct large, chemically consistent structural libraries without manual drawing, we implemented a Python/RDKit workflow (Figure 9) for automated generation of meso-substituted porphyrinoid SMILES strings. This automated batch generator is distinct from the interactive Site 1 interface described later: the batch module is a stochastic, scaffold-specific, meso-only generator, whereas interactive Site 1 is a user-directed constructor that can expose predefined meso and β attachment positions. The batch workflow contains three scaffold-specific generators together with a shared module defining substituent libraries and molecular assembly routines. Each generator imports a macrocyclic MOL template, sanitizes the structure, computes two-dimensional coordinates, identifies substitution-accessible meso carbons using scaffold-specific graph-connectivity rules, and then stochastically attaches substituent fragments to generate canonical SMILES. Accepted entries are exported as canonical SMILES together with substituent labels, enabling direct use in cheminformatics pipelines or as input to external computational workflows (Figure 9).

Figure 9 summarizes the automated batch SMILES workflow step by step. The pipeline begins by importing a scaffold-specific MOL template (porphine, chlorophyll-like, or bacteriochlorophyll-like). The template is sanitized and assigned 2D coordinates, after which accessible meso carbons are identified using the scaffold-specific connectivity rules above. Substituent fragments are then sampled from a single electronic category, attached to the selected meso- or β-sites, and re-sanitized. Chemically invalid or duplicate structures are rejected; accepted molecules are converted to canonical SMILES and exported with substituent metadata for downstream Site 2/Site 3 processing.

#### 4.1.1. Core Templates and Substituent Identification

Three macrocyclic scaffolds were employed:

- Free-base porphine (Porphine.mol): All four meso carbons were treated as accessible sites. These meso carbons were identified as ring-bound carbon atoms that are bonded to exactly two other atoms in the molecular graph (i.e., unsubstituted meso positions).
- Chlorophyll-like core (Chl.mol): Ring carbons that already carry additional substituents (bonded to more than two neighboring atoms in the molecular graph) were regarded as already substituted and excluded. Remaining unsubstituted ring carbons bonded to exactly two neighbors constituted the accessible meso pool.
- Bacteriochlorophyll-like core (BChl.mol): The chlorophyll-like exclusion rule was extended by additionally removing meso carbons adjacent to gem-dimethyl-bearing ring centers. Dimethyl groups were detected as two terminal methyl carbons (each bonded to only one neighboring atom) attached to a ring carbon that already carries more than two neighbors.

After import, each template was sanitized and assigned two-dimensional coordinates using RDKit prior to substituent attachment. In RDKit, sanitization is a sequence of molecular-graph validation and normalization operations: it checks valence and bonding consistency, assigns aromaticity and conjugation, performs kekulization and ring-related checks, and rejects structures that cannot be represented as chemically valid RDKit molecules. In this workflow, sanitization is applied after template import and after each fragment attachment; a failed sanitization causes the trial structure to be rejected rather than passed downstream.

#### 4.1.2. Substituent Libraries

Substituents were grouped into three electronic categories: (1) electron-withdrawing, (2) electron-donating, and (3) conjugated groups. Each category contained a predefined set of R-group fragments encoded as SMILES strings (13, 8, and 9 fragments, respectively). Within any single generated structure, all substituents were drawn from one category only, so that each output file contains an electronically homogeneous subset of meso-substituted porphyrinoids.

#### 4.1.3. Generation Algorithm

Structure assembly followed a rejection-sampling protocol implemented in RDKit:

- For each category, 500 unique structures were targeted by default (1500 per scaffold).
- In each trial, an integer k ∈ {1, 2, 3, 4} was drawn to specify the number of meso positions to functionalize.
- k distinct meso sites and k substituents from the active category were selected at random.
- Substituent fragments were merged onto the core via single-bond formation using an editable molecular graph (RWMol). After each attachment, the intermediate was sanitized; chemically invalid trials were discarded.
- Accepted structures were converted to canonical SMILES and retained only if not previously recorded within the same category.

### 4.2. System Architecture

#### 4.2.1. Task-Oriented User Experience Design

The website is designed to enable users to predict the properties of porphyrin molecules within a relatively short time frame. A key advantage of the platform is that it supports property prediction for both existing and hypothetical porphyrin molecules, and, through AI-assisted computational approaches, the system overcomes many of the limitations associated with traditional experimental methods. The design philosophy here is task-oriented, partitioning the porphyrin design and property prediction workflow into four distinct tasks, each of which can be accessed and modified at any stage. The four tasks consist of (1) substituent selection at different positions with automatic SMILES generation, (2) geometric optimization, (3) orbital energy prediction, and (4) simulation and visualization of the spectrum. While researchers are using the website features, the webpage shows only the step where the user is currently located, making each step manageable without complication. Through this step-by-step approach, users will be able to focus on the current task, inspect intermediate outputs, and adjust the workflow at any stage, enabling a visualizable, traceable, and correctable user experience. In summary, the entire website is constructed as a structure of “layer-by-layer progression, streamlined operations, and clear error localization.”

The website adopts modular multi-site architecture. Specifically, the entire website consists of four Flask applications: Site 1, Site 2, Site 3, and Site 4, which communicate through lightweight JSON data structures. Each site implements one function in the design concept. Site 1 provides two explicit workflows. In interactive mode, the user selects the porphyrin macrocycle core, substituent positions (meso and/or β), and substituent groups; the platform then assembles the specified structure, verifies chemical validity, and returns canonical SMILES. In batch mode, the code imports a scaffold MOL template, identifies meso sites by graph-connectivity rules, samples the number and identity of substituents, attaches fragments, rejects invalid or duplicate structures, and exports canonical SMILES plus metadata. Site 2 receives SMILES and performs RDKit [24] and MMFF94 [25] geometry optimization for optional 2D/3D visualization and structure export. The SMILES generated by Site 1 are also transmitted to Site 3; Site 3 does not require MMFF94-optimized geometries as input, because it uses 2D ECFP6 fingerprints derived from SMILES together with the previously trained AutoGluon model [11,12,13]. Finally, based on the orbital energies predicted by Site 3, Site 4 performs spectrum calculation and visualization based on the Gouterman four-orbital model. The front end uses HTML and CSS to maintain a consistent interface, and switching between sites is entirely achieved through AJAX; therefore, users do not need to reload the page or manually download/upload files during operation.

In the user interaction part, the system provides clear operational feedback for each task node. For erroneous operations, the website reports the reason for failure at the corresponding site. In Site 1, substituent combinations immediately generate SMILES and perform structural legality verification, and errors are highlighted in the interface. SMILES can be automatically transmitted to Site 2 through hidden POST fields without manual copying. During geometry optimization, the system outputs convergence information so that users can determine whether MMFF94 optimization has converged. For orbital energies, results are shown in a table that also serves as input for Site 4. In batch mode, independent task status bars are generated for each molecule. For example, if molecule #17 fails sanitization because an assigned substituent produces an impossible valence at a meso carbon, Site 1 flags that entry as invalid; the user can return to the substituent selector, replace the offending group (or reduce the substitution pattern), regenerate the SMILES, and resubmit only the corrected molecule without restarting the entire batch.

The amount of data transmitted between sites is also strictly controlled to be minimal, such as SMILES strings, orbital energy level parameters, or very small status markers. Therefore, network overhead is extremely small, and delays will mainly come from the computation itself rather than the network or interface loading. These features not only make the user’s single-use experience smoother but also allow batch task computation and analysis to run smoothly without increasing system burden. Taken together, the system ultimately realizes a molecular design and spectral analysis platform centered on smooth task processing and operational efficiency.

#### 4.2.2. Constructing an Interactive and Customizable Spectrum Display Interface

**Figure 10:**
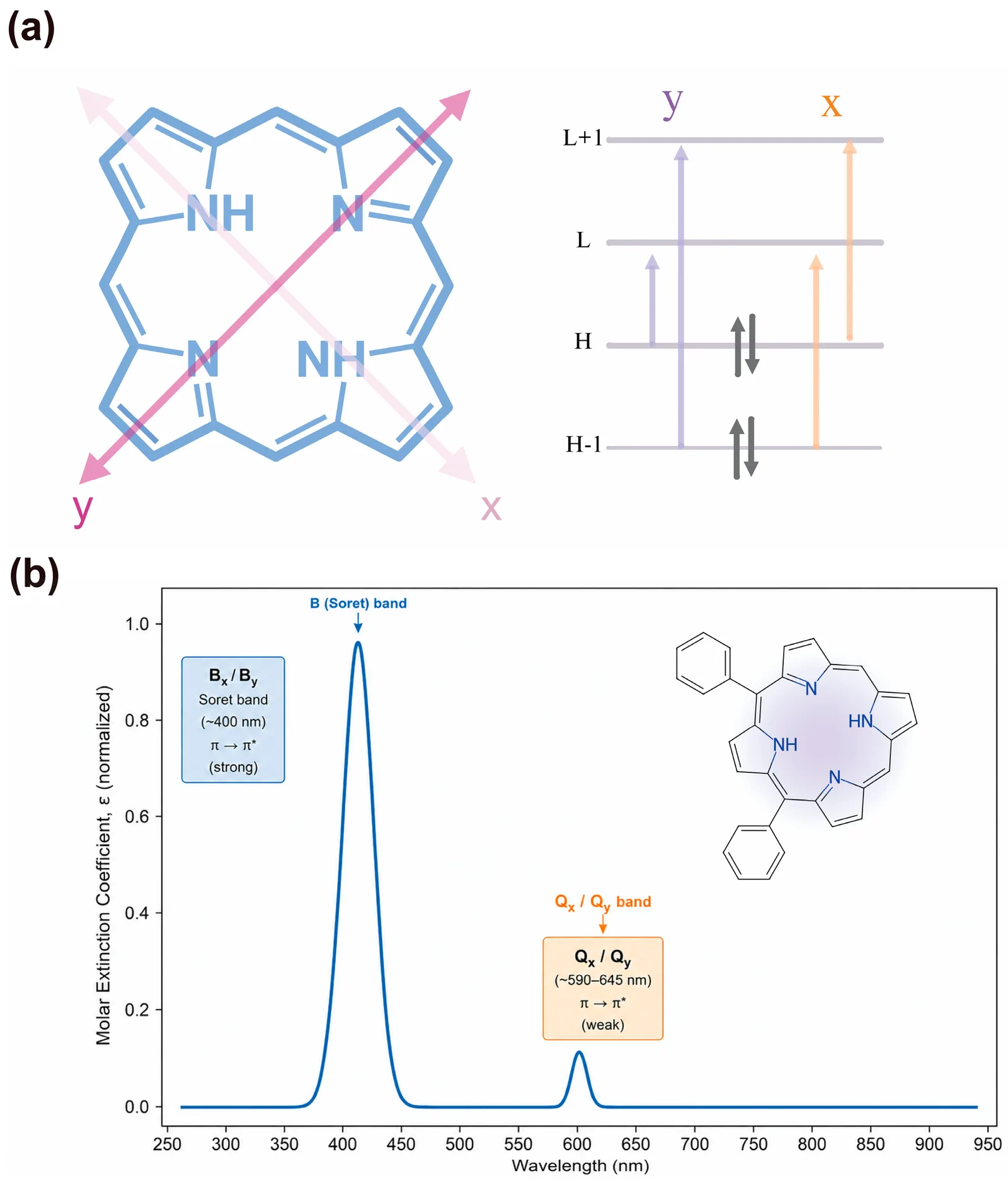
Gouterman four-orbital model for tetrapyrrole B and Q bands: (**a**) Frontier orbitals (H − 1/H and L/L + 1; a1u/a2u and eg nodal patterns) and x/y-polarized one-electron promotions that mix to give the B and Q states. (**b**) Representative normalized absorption spectrum of 5,10-diphenylporphyrin showing the intense B (Soret) and weak Q bands. In panel (**b**), π → π* denotes an electronic promotion from a bonding π orbital to an antibonding π* orbital (the asterisk marks the antibonding level).

Spectral visualization and exportable data tables are the core outputs from this system. To display the predicted data, the system uses a high-performance front-end graphics library to plot spectra containing hundreds of data points. The sampling interval is fixed at 1 nm over 250–950 nm, and downloadable tables contain the columns “Wavelength (nm)” and “Normalized absorbance”. Importantly, wavelength is the physical wavelength in nanometers and is not normalized; only the plotted intensity (y-axis) is normalized relative to the maximum value. The interface annotates the energy, wavelength, and normalized intensity of the four electronic peaks (By, Bx, Qy, and Qx). Peak values alone are not sufficient to judge reliability: the current model does not provide calibrated predictive uncertainty or an automated applicability-domain score, so the interface must not present the output as self-validating. Users are therefore encouraged to inspect band positions/intensities together with chemical context and, where possible, independent experimental or higher-level computational references. An interactive cursor readout displays wavelength and normalized absorbance at any point along the curve. Figure 10 summarizes how the four frontier orbitals and their configuration interaction give rise to the intense B and weak Q bands in the Gouterman model.

On the server side, spectral data are generated through NumPy [26] and Pandas [27] and then returned to the front end in JSON format. When the spectrum is recalculated, users do not need to refresh the page. If the full width at half-maximum (FWHM) or other correction parameters need modifications, only a new small data array needs to be transmitted, and the front end can immediately update the chart. For batch tasks, the system automatically aligns all spectra by wavelength index and merges them into a multi-column CSV table that facilitates horizontal analysis.

The Site 4 spectral-reconstruction procedure builds on the Gouterman four-orbital formalism described in our previous Scientific Reports study and on the transition-dipole treatment implemented in the GOUTERMAN module [12,18]. The orbital-pairing relations, configuration-interaction treatment, empirical energy corrections, oscillator-strength expressions, and Gaussian broadening procedure described below represent the specific numerical implementation adopted in BeyondPhoton Site 4.

The four one-electron promotion energies are

(1) $$\Delta E\left(a_{2u}\to e_{gy}\right)=E\left(e_{gy}\right)-E\left(a_{2u}\right)\;\Delta E\left(a_{1u}\to e_{gx}\right)=E\left(e_{gx}\right)-E\left(a_{1u}\right)\;\Delta E\left(a_{2u}\to e_{gx}\right)=E\left(e_{gx}\right)-E\left(a_{2u}\right)\;\Delta E\left(a_{1u}\to e_{gy}\right)=E\left(e_{gy}\right)-E\left(a_{1u}\right)$$

For the y- and x-polarized configuration pairs, the average and difference energies are

(2) $$E_{avg}^{y}=\frac{1}{2}\left[\Delta E\left(a_{2u}\to e_{gy}\right)+\Delta E\left(a_{1u}\to e_{gx}\right)\right]\;\Delta E^{y}=\left|\Delta E\left(a_{2u}\to e_{gy}\right)-\Delta E\left(a_{1u}\to e_{gx}\right)\right|$$

(3) $$E_{avg}^{x}=\frac{1}{2}\left[\Delta E\left(a_{2u}\to e_{gx}\right)+\Delta E\left(a_{1u}\to e_{gy}\right)\right]\;\Delta E^{x}=\left|\Delta E\left(a_{2u}\to e_{gx}\right)-\Delta E\left(a_{1u}\to e_{gy}\right)\right|$$

Configuration interaction with coupling energy $E_{CI}$ mixes each nearly degenerate pair. The mixing angles are defined as

(4) $$\eta^{y}=\frac{1}{2}arctan\left(\frac{\Delta E^{y}}{2E_{CI}}\right)\;\eta^{x}=\frac{1}{2}arctan\left(\frac{\Delta E^{x}}{2E_{CI}}\right)$$

The B- and Q-band transition energies, expressed in electronvolts, are then calculated as

(5) $$E_{B_{y}}=E_{avg}^{y}+\sqrt{E_{CI}^{2}+{\left(\frac{\Delta E^{y}}{2}\right)}^{2}}+E_{cor}-\delta$$

(6) $$E_{Q_{y}}=E_{avg}^{y}-\sqrt{E_{CI}^{2}+{\left(\frac{\Delta E^{y}}{2}\right)}^{2}}+E_{cor}-\delta$$

(7) $$E_{B_{x}}=E_{avg}^{x}+\sqrt{E_{CI}^{2}+{\left(\frac{\Delta E^{x}}{2}\right)}^{2}}+E_{cor}-\delta$$

(8) $$E_{Q_{x}}=E_{avg}^{x}-\sqrt{E_{CI}^{2}+{\left(\frac{\Delta E^{x}}{2}\right)}^{2}}+E_{cor}-\delta$$

Here, $E_{cor}$ is the global empirical energy correction, and δ is the additional empirical correction parameter used in Site 4. As listed in Table 7, the default values are $E_{CI}=0.4$ eV, $E_{cor}=0.4$ eV, and δ=0.8 eV.

The relative oscillator strengths are calculated using the transition-dipole framework implemented in the GOUTERMAN module:

(9) $$f_{B_{y}}=\frac{2}{3}\left(1+cos2\eta^{y}\right)\left[E_{avg}^{y}+\sqrt{E_{CI}^{2}+{\left(\frac{\Delta E^{y}}{2}\right)}^{2}}\right]\left(\frac{TDM_{0}^{2}}{27.2114}\right)$$

(10) $$f_{Q_{y}}=\frac{2}{3}\left(1-cos2\eta^{y}\right)\left[E_{avg}^{y}-\sqrt{E_{CI}^{2}+{\left(\frac{\Delta E^{y}}{2}\right)}^{2}}\right]\left(\frac{TDM_{0}^{2}}{27.2114}\right)$$

(11) $$f_{B_{x}}=\frac{2}{3}\left(1+cos2\eta^{x}\right)\left[E_{avg}^{x}+\sqrt{E_{CI}^{2}+{\left(\frac{\Delta E^{x}}{2}\right)}^{2}}\right]\left(\frac{TDM_{0}^{2}}{27.2114}\right)$$

(12) $$f_{Q_{x}}=\frac{2}{3}\left(1-cos2\eta^{x}\right)\left[E_{avg}^{x}-\sqrt{E_{CI}^{2}+{\left(\frac{\Delta E^{x}}{2}\right)}^{2}}\right]\left(\frac{TDM_{0}^{2}}{27.2114}\right)$$

The default transition-dipole parameter is $TDM_{0}=3.2$. The peak amplitudes used for spectral construction are

$$\varepsilon_{i}=\frac{f_{i}}{K\cdot FWHM_{i}}$$

where $K=4.33\times 10^{-9}$, and $FWHM_{i}$ represents the user-adjustable full width at half-maximum for the $B_{y}$, $B_{x}$, $Q_{y}$, and $Q_{x}$ transitions, as listed in Table 7.

Each transition is broadened over a wavelength range of 250–950 nm, using a 1 nm grid, by applying a Gaussian function in the energy domain:

(13) $$I_{i}\left(E\right)=\varepsilon_{i}exp\left[-\frac{{\left(E-E_{i}\right)}^{2}}{2\sigma_{i}^{2}}\right]$$

$$\sigma_{i}=\frac{FWHM_{i}\cdot hc}{2.35482}$$

where

$$E=\frac{1240}{\lambda \;\left(nm\right)}\;\left(eV\right)$$

$$hc=1.23984\times 10^{-4}\;\left(eV\cdot cm\right)$$

Here, E and $E_{i}$ are expressed in electronvolts, λ is expressed in nanometers, and $FWHM_{i}$ is entered in cm^−1^. The final continuous spectrum is obtained by summing the four Gaussian components:

$$I_{total}\left(E\right)=I_{B_{y}}\left(E\right)+I_{B_{x}}\left(E\right)+I_{Q_{y}}\left(E\right)+I_{Q_{x}}\left(E\right)$$

For visualization, the resulting spectrum y-axis is normalized to the range from 0 to 1, while the horizontal axis remains the physical wavelength scale in nanometers.

#### 4.2.3. Overview of the System Reconstruction Process

The system reconstruction process adopts the design concept of an Activity Diagram [28]. In this framework, action nodes represent individual computational steps, including SMILES generation, molecular embedding, geometry optimization, orbital energy prediction, and spectral synthesis. Decision nodes are used to denote validation stages, such as structural legality checks, convergence of geometry optimization, and successful generation of molecular orbitals. Directed arrows indicate the flow of tasks between these nodes, ensuring that the user’s operational pathway remains clear, logical, and fully traceable.

The reconstruction process was primarily carried out by two teams. Domain experts in porphyrin photophysics and photochemistry were responsible for defining scientific requirements, including representative substituents, orbital energy level ranges, and model correction strategies. Software development teams translated these requirements into concrete implementations, encompassing API design, data structures, front-end interaction logic, and rendering specifications. The reconstruction workflow involved systematic analysis of legacy scripts, development of user personas, creation of low-fidelity interface prototypes, iterative testing and refinement, and the integration of error recovery mechanisms and data export functionalities. The outcome is a robust and fully operational platform in which scientific constraints and interface design are tightly integrated, achieving high stability and a low barrier to use.

## 5. Conclusions

In this work, we developed BeyondPhoton, a modular web- and software-based platform for porphyrin molecular design and UV–Vis–NIR spectral prediction. By integrating batch molecular design, structure preparation, frontier-orbital energy prediction, and electronic spectral simulation into a continuous workflow, the platform addresses common fragmentation in conventional computational chemistry tools. The four interconnected modules enable automated data transfer while preserving transparency and independent usability at each step. Users can design porphyrin derivatives by visual selection of peripheral substituents or direct SMILES input, inspect and download optimized 2D/3D structures, predict key molecular orbital energies, and generate intensity-normalized absorption spectra with annotated B_y_, B_x_, Q_y_, and Q_x_ transitions. Batch processing, interactive visualization, and exportable data formats support rapid screening and downstream analysis. Future development will focus on experimental spectral calibration, improved predictive accuracy, explicit treatment of vibronic and solvent effects, MCD-enabled validation, and extension toward metalloporphyrins and broader tetrapyrrole families within carefully expanded training domains.

## Figures and Tables

**Table 1:** Data fields processed by Site 2.

Field	Type	Description
Input SMILES	String	Original SMILES input by users
Canonical SMILES	String	Structure standardized by RDKit
InChI key	String	Unique molecular identifier
Status	Status marker	Processing success or failure
MMFF energy	Numerical value	MMFF94 optimized energy value (if successful)
Mol block	Text	RDKit MolBlock for 3D optimization and rendering

**Table 2:** Available substituent categories in Site 1.

Substituent Category	Examples
Halogen	Fluoro (F), Chloro (Cl), Bromo (Br), Iodo (I)
Alkyl	Methyl (Me), Ethyl (Et), Isopropyl (i-Pr), tert-Butyl (t-Bu)
Alkenyl	Vinyl (Vinyl), Allyl (Allyl), Styryl (Sty), Butadienyl (Butadienyl)
Alkynyl	Alkyne (C≡C–)
Oxygen-containing groups	Hydroxy (OH), Methoxy (OMe)
Nitrogen-containing groups	Amino (NH_2_), Dimethylamino (NMe_2_), Nitro (NO_2_), Cyano (CN)
Sulfur-containing groups	Thiomethyl (SMe)
Carbonyl-containing groups	Carbonyl ketone (COR), Carbonyl aldehyde (CHO), Carboxylic acid (COOH)
Fluoroalkyl	Trifluoromethyl (CF_3_)
Aryl	Phenyl (Ph), Ethynylphenyl (Ethynyl-Ph)
Heteroaryl	Thiophene (Th), Furan (Fu), Pyridine (Py)

**Table 3:** Prior validation metrics for the Site 3 frontier-orbital energy model (metal-free porphyrins, chlorins, and bacteriochlorins; n = 1437) reported in the Scientific Reports study [12].

Dataset	R^2^	MAE (eV)	RMSE (eV)
Validation	0.985	0.077	0.133
Held-out test	0.936	0.168	0.287

**Table 4:** Peak information reported by Site 4.

Peak Label	Wavelength (nm) ^a^	Normalized Intensity	Description
B_y_	Calculated value	Normalized value	B band corresponding to y-polarized transition
Q_y_	Calculated value	Normalized value	Q band corresponding to y-polarized transition
B_x_	Calculated value	Normalized value	B band corresponding to x-polarized transition
Q_x_	Calculated value	Normalized value	Q band corresponding to x-polarized transition

^a^ The wavelength values represent actual wavelengths in nanometers (not normalized), while the intensity values (ε) are normalized such that the highest peak among the four transitions has a value of 1.0, with all other peaks scaled proportionally.

**Table 5:** Absolute Soret/B and Q-band peak positions for TPP from the experimental (Real), BeyondPhoton-predicted, and DFT-simulated normalized spectra in Figure 8. Photon energies were obtained as E (eV) = 1239.84/λ (nm). Δλ is relative to the experimental Soret maximum at 396 nm for B-band peaks.

Source	B/Soret λ (nm)	B/Soret E (eV)	Q-Band λ (nm)	Q-Band E (eV)	Δλ_B (nm)
Experiment (Real)	396	3.131	563 ^a^	2.202	—
BeyondPhoton	405	3.061	585	2.119	+9
DFT simulation	404	3.069	583	2.126	+8

^a^ The experimental Q region contains multiple weak features (including intensity near ~488 nm attributed to vibronic/solvent contributions). The 563 nm value is the strongest local maximum between 520 and 600 nm in the experimental trace and is listed for band-level comparison only.

**Table 6:** Representative journals in porphyrin photophysics, photochemistry, and related fields, grouped by publisher.

Publisher	Example Journals in Relevant Fields
MDPI	*Molecules, Photochem, Materials*
American Chemical Society (ACS)	*J. Am. Chem. Soc., J. Phys. Chem., Inorg. Chem., J. Org. Chem.*
Royal Society of Chemistry (RSC)	*Chem. Sci., Phys. Chem. Chem. Phys., RSC Adv.*
Wiley	*Angew. Chem. Int. Ed., Chem. Eur. J.*
Elsevier	*J. Photochem. Photobiol., J. Chem. Phys.*
Springer Nature	*Nat. Chem., Sci. Rep.*

**Table 7:** Spectrum calculation parameters.

Parameter	Default Value	Description
Spectral range	250–950 nm	Spectral calculation interval uniformly used by the system.
Sampling step length	1 nm	Internally fixed parameter used for all spectra.
E_CI	0.4 eV	Configuration interaction energy parameter used in the four-orbital model (modifiable in the program).
E_cor	0.4 eV	Global empirical energy correction value (modifiable within the program).
Correction δ	0.8	Empirical energy offset δ (eV) subtracted in Equations (5)–(8); default 0.8 eV in Site 4.
FWHM (B_y_, B_x_, Q_y_, Q_x_)	See website interface	Half-width at half-maximum corresponding to the four absorption peaks (freely adjustable by the user).
Output files	PNG, CSV, ZIP	Downloadable formats provided by the system.

## Data Availability

Two software tools developed in this study are openly distributed. The automated SMILES generation process is available at https://github.com/gwofanzhang/X2SMILES (accessed on 23 August 2026). BeyondPhoton Software is available at https://github.com/gwofanzhang/BeyondPhoton (accessed on 23 August 2026); BeyondPhoton Web is available at https://www.beyondphoton.com/ (accessed on 23 August 2026), free of charge.

## Abbreviations

The following abbreviations are used in this manuscript:

**SMILES**	Simplified Molecular Input Line Entry System
**UV-Vis-NIR**	Ultraviolet–Visible–Near-Infrared
**HOMO**	Highest Occupied Molecular Orbital
**LUMO**	Lowest Unoccupied Molecular Orbital
**MMFF94**	Merck Molecular Force Field version 94
**FWHM**	Full Width at Half-Maximum
**DFT**	Density Functional Theory
**TDDFT**	Time-Dependent Density Functional Theory
**LLM**	Large Language Model
**JSON**	JavaScript Object Notation
**API**	Application Programming Interface
**CSVs**	Comma-Separated Values
**PNG**	Portable Network Graphics
